# Supplementary material for: Quantifying the Consistency of Scientific Databases
Source: PLoS One. 2015 May 18;10(5):e0127390. doi: 10.1371/journal.pone.0127390 (PMC4436008; doi:10.1371/journal.pone.0127390)
Supplement: S1 File — Node degree and clustering profiles and distributions of all the considered networks, along with other network statistics. See Methods for interpretation and details on computation. (PDF) [file pone.0127390.s001.pdf]

Quantifying the Consistency of Scientific Databases

(Supporting Information File S1)

Lovro Šubelj[1], Marko Bajec[1], Biljana Mileva Boshkoska[2],
Andrej Kastrin[2], Zoran Levnajić[1,2]

[1] Faculty of Computer and Information Science, University of Ljubljana, Slovenia
[2] Faculty of Information Studies in Novo mesto, Novo mesto, Slovenia
[*] Correspondence and requests for materials should be addressed to Lovro Šubelj
(lovro.subelj@fri.uni-lj.si)

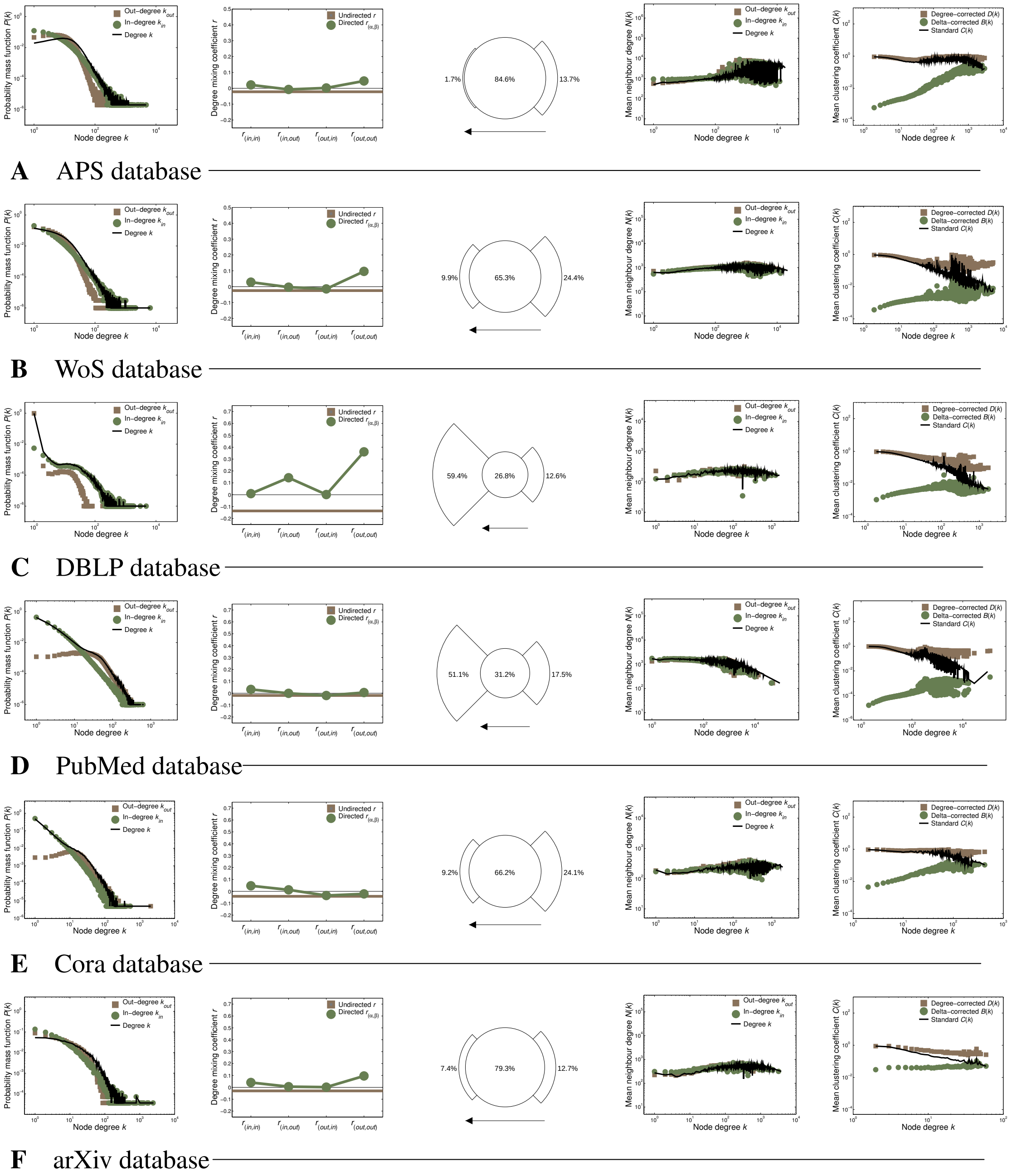

Figure A: **Degree and clustering graphical profiles.** Node degree and clustering profiles and distributions of P→P networks (first and second column), A↔A networks (third and fourth column) and A−A networks (last column). See Methods for interpretation and details on computation.

Table B1: **Continuation of network measures 1.** Degree statistics for all 18 considered networks.

		Degree distributions					Degree mixing			
Type	Database	$\langle k \rangle$	γ	γ_{in}	γ_{out}	r	$r_{(in,in)}$	$r_{(in,out)}$	$r_{(out,in)}$	$r_{(out,out)}$
P$\rightarrow$P	APS	20.85	2.28	2.33	3.10	-0.02	0.02	-0.01	0.00	0.05
	WoS	9.97	2.67	2.39	3.67	-0.02	0.03	-0.00	-0.01	0.10
	DBLP	2.05	1.80	1.75	2.82	-0.14	0.01	0.14	0.00	0.36
	PubMed	6.42	2.09	2.91	1.89	-0.02	0.03	0.00	-0.02	0.01
	Cora	6.21	2.30	2.74	2.34	-0.04	0.05	0.01	-0.04	-0.02
	arXiv	25.41	1.98	2.12	2.35	-0.03	0.04	0.01	0.00	0.10
A$\leftrightarrow$A	APS	311.00	1.46	1.53	1.56	0.30	0.27	0.29	0.34	0.40
	WoS	86.31	1.63	1.71	1.76	-0.01	0.00	0.00	-0.01	0.01
	DBLP	29.46	1.79	1.97	1.86	-0.04	0.01	-0.01	-0.03	0.00
	PubMed	37.31	1.85	2.08	1.85	-0.02	-0.02	-0.02	-0.02	-0.02
	Cora	54.09	1.70	1.78	1.87	0.02	0.06	0.06	0.07	0.08
	arXiv	99.59	1.56	1.68	1.67	-0.04	0.02	0.06	0.01	0.09
A$-$A	APS	34.00	1.75	-	-	0.57	-	-	-	-
	WoS	11.15	2.25	-	-	0.00	-	-	-	-
	DBLP	8.56	2.41	-	-	0.10	-	-	-	-
	PubMed	20.21	2.12	-	-	-0.01	-	-	-	-
	Cora	11.13	2.17	-	-	0.29	-	-	-	-
	arXiv	4.15	3.45	-	-	0.13	-	-	-	-

Table B2: **Continuation of network measures 2.** Clustering and diameter statistics for all 18 considered networks.

		Clustering distributions			Clustering mixing			Diameter
Type	Database	$\langle c \rangle$	$\langle b \rangle$	$\langle d \rangle$	r_c	r_b	r_d	δ_{90}
P→P	APS	0.24	$0.08 \cdot 10^{-2}$	0.28	0.12	0.43	0.33	6.91 ± 0.05
	WoS	0.15	$0.02 \cdot 10^{-2}$	0.18	0.22	0.51	0.44	8.59 ± 0.11
	DBLP	0.00	$0.00 \cdot 10^{-2}$	0.00	0.49	0.66	0.68	8.74 ± 0.01
	PubMed	0.03	$0.00 \cdot 10^{-2}$	0.04	0.05	0.41	0.27	9.02 ± 0.12
	Cora	0.11	$0.03 \cdot 10^{-2}$	0.12	0.07	0.43	0.33	7.87 ± 0.06
	arXiv	0.31	$0.26 \cdot 10^{-2}$	0.37	0.13	0.51	0.37	6.45 ± 0.16
A↔A	APS	0.39	$0.52 \cdot 10^{-2}$	0.43	0.38	0.71	0.74	4.32 ± 0.06
	WoS	0.31	$0.05 \cdot 10^{-2}$	0.34	0.06	0.12	0.19	4.79 ± 0.03
	DBLP	0.41	$0.41 \cdot 10^{-2}$	0.45	-0.05	0.17	0.25	5.10 ± 0.05
	PubMed	0.28	$0.01 \cdot 10^{-2}$	0.31	0.11	0.29	0.28	4.94 ± 0.03
	Cora	0.46	$0.66 \cdot 10^{-2}$	0.51	-0.01	0.34	0.18	4.79 ± 0.04
	arXiv	0.47	$0.70 \cdot 10^{-2}$	0.53	-0.08	0.22	0.15	4.43 ± 0.06
A−A	APS	0.62	$0.76 \cdot 10^{-2}$	0.69	0.25	0.87	0.48	8.38 ± 0.09
	WoS	0.60	$0.08 \cdot 10^{-2}$	0.66	0.24	0.91	0.36	6.89 ± 0.06
	DBLP	0.63	$0.22 \cdot 10^{-2}$	0.70	0.19	0.84	0.34	7.81 ± 0.03
	PubMed	0.67	$0.01 \cdot 10^{-2}$	0.74	0.14	0.46	0.22	5.86 ± 0.09
	Cora	0.68	$1.66 \cdot 10^{-2}$	0.75	0.19	0.85	0.31	7.18 ± 0.08
	arXiv	0.48	$2.89 \cdot 10^{-2}$	0.57	0.22	0.64	0.40	9.31 ± 0.07
